# Functional Connectivity and Tuning Curves in Populations of Simultaneously Recorded Neurons

**DOI:** 10.1371/journal.pcbi.1002775

**Published:** 2012-11-15

**Authors:** Ian H. Stevenson, Brian M. London, Emily R. Oby, Nicholas A. Sachs, Jacob Reimer, Bernhard Englitz, Stephen V. David, Shihab A. Shamma, Timothy J. Blanche, Kenji Mizuseki, Amin Zandvakili, Nicholas G. Hatsopoulos, Lee E. Miller, Konrad P. Kording

**Affiliations:** 1 Redwood Center for Theoretical Neuroscience, University of California, Berkeley, Berkeley, California, United States of America; 2 Department of Physiology, Northwestern University, Chicago, Illinois, United States of America; 3 Department of Biomedical Engineering, Northwestern University, Evanston, Illinois, United States of America; 4 Department of Neuroscience, Baylor College of Medicine, Houston, Texas, United States of America; 5 Oregon Hearing Research Center, Oregon Health and Science University, Portland, Oregon, United States of America; 6 Department of Electrical Engineering, University of Maryland College Park, College Park, Maryland, United States of America; 7 Center for Molecular and Behavioral Neuroscience, Rutgers University, Newark, New Jersey, United States of America; 8 Department of Neuroscience, Albert Einstein College of Medicine, Bronx, New York, United States of America; 9 Department of Organismal Biology and Anatomy, University of Chicago, Chicago, Illinois, United States of America; 10 Department of Physical Medicine and Rehabilitation, Northwestern University and Rehabilitation Institute of Chicago, Chicago, Illinois, United States of America; University of Oxford, United Kingdom

## Abstract

How interactions between neurons relate to tuned neural responses is a longstanding question in systems neuroscience. Here we use statistical modeling and simultaneous multi-electrode recordings to explore the relationship between these interactions and tuning curves in six different brain areas. We find that, in most cases, functional interactions between neurons provide an explanation of spiking that complements and, in some cases, surpasses the influence of canonical tuning curves. Modeling functional interactions improves both encoding and decoding accuracy by accounting for noise correlations and features of the external world that tuning curves fail to capture. In cortex, modeling coupling alone allows spikes to be predicted more accurately than tuning curve models based on external variables. These results suggest that statistical models of functional interactions between even relatively small numbers of neurons may provide a useful framework for examining neural coding.

## Author Summary

The number of simultaneous neurons that electrophysiologists can record is growing rapidly, and a central goal of computational neuroscience is to develop statistical methods that can make sense of this growing data. Here we present a unified statistical analysis of 10 different datasets recorded from several different species and brain areas. We show how functional interactions between neurons may be used to predict spiking in each of these different areas, and find that, in many cases, modeling interactions between a small number of neurons yields better spike predictions than modeling each neuron's relationship to the outside world using tuning curves. Although these statistical results cannot be linked to specific network architectures, since the measured interactions between neurons are purely functional rather than anatomical, they suggest that modeling interactions between neurons will be a useful approach to understanding neural coding as electrophysiologists record from increasing numbers of neurons.

## Introduction

One of the central tenets of systems neuroscience is that the functional properties of neurons, such as receptive fields and tuning curves, arise from the inputs that each neuron receives from pre-synaptic neurons. Over the past few decades, a number of experimental techniques have been developed to study exactly how interactions between neurons determine receptive field structure, including *in vivo* intracellular or paired recording [1]–[6] and pharmacological or electrophysiological interventions [7]–[10]. As electrophysiologists record from increasing numbers of neurons simultaneously [11]–[13], statistical approaches that estimate interactions between neurons have the potential to explain the functional properties of neurons as network effects using only passive spike observations [for review see 14].

To understand how interactions between neurons drive neural activity, recent model-based statistical methods attempt to predict the activity of each neuron based on the activity of other simultaneously observed neurons in addition to any external variables, such as the orientation of a visual stimulus or the direction of hand movement [15]–[19]. This type of inferential approach provides estimates of potential interactions between neurons and allows us to assess how much external variables or interactions between neurons may have contributed to the observed spiking. It is important to note that these models provide only an approximation to the true underlying network structure. Since the vast majority of pre-synaptic inputs to any given neuron are unobserved, the interactions that these approaches describe reflect many different factors including common input in addition to direct and indirect synaptic connections [14], [20]. However, due to the fact that neurons are not independent, these models can improve both encoding accuracy (how well neural responses can be predicted) as well as decoding accuracy (how well external variables can be predicted from neural responses).

Statistical models of interactions between neurons have been used to describe many different aspects of multi-electrode data in retina [21], LGN [22], primary visual cortex [23], [24], motor cortices [17], [25], [26], and hippocampus [27], [28]. Here we present a unified analysis of data from six different brain areas with a particular view towards three questions: 1) How are estimated interactions between neurons related to apparent tuning properties?, 2) How do traditional tuning curve models change as interactions between neurons are included in the model? and 3) How does our ability to predict and decode neural activity improve as increasing numbers of simultaneously recorded neurons are observed?

To make our analysis as broad as possible, we collected ten multi-electrode spike train datasets with at least 30 simultaneously recorded neurons. Datasets were obtained from six different brain areas across four different species performing a variety of tasks. By modeling typical tuning curves for neurons in each area as well as interactions between neurons we determine how much these two factors contribute to spike prediction. We find that including information about the activity of other observed neurons improves both spike prediction and decoding accuracy substantially. By capturing noise correlations and unmodeled features of the external world models of interactions between even a relatively small number of recorded neurons can complement and, in some cases, surpass, models of tuning curves alone.

## Results

**Figure 1:**
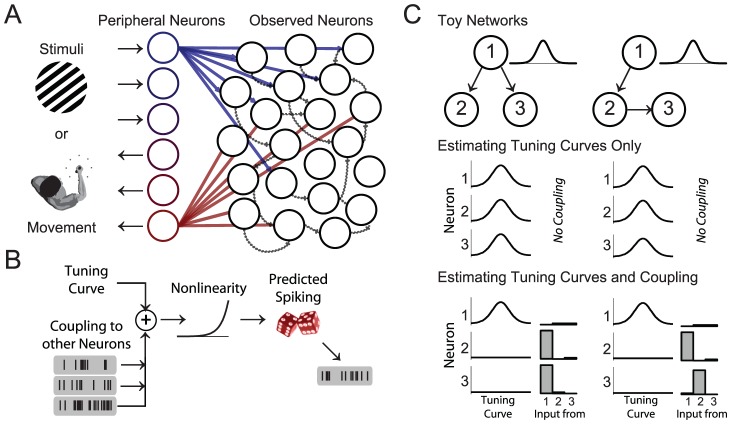
Functional connectivity and tuning curves. A) Schematic illustrating how tuning properties may be related to functional connectivity. The tuning properties of observed neurons (black) are a direct result of input they receive from peripheral neurons (blue and red). Even when observed neurons do not have a direct relationship to external variables, each neuron may have apparent tuning caused by the input it receives from peripheral neurons and shaped by interactions with other observed neurons. B) A linear-nonlinear-Poisson model that includes both tuning properties as well as interactions or coupling between neurons. The firing rate of each neuron is modeled as a weighted sum of external variables as well as the activities of other observed neurons passed through an exponential nonlinearity. C) A toy example where tuning properties are explained away using coupling. In two simulated networks only neuron 1 is directly related to the external world (with Gaussian tuning). However, neurons 2 and 3 have tuning due to the input they receive from neuron 1 (middle). If these interactions are estimated, then coupling can fully explain the observed tuning (bottom).

Although neurons are often characterized by how their firing rate relates to external stimuli or movement variables, the functional properties of most neurons are byproducts of the input they receive from other neurons (Fig. 1A). By modeling typical tuning curves as well as coupling between neurons we aim to determine how well each of these factors explains spiking (Fig. 1B). We fit three, time-instantaneous, generalized linear models (GLMs) to recorded spike trains from 10 different datasets and attempt to predict spiking given: 1) external variables, 2) the activity of other observed neurons, or 3) both external variables and the activity of other observed neurons. After fitting these models to spike data the estimated parameters correspond to a typical tuning curve model, a phenomenological model of interactions between neurons, and a full model that allows functional interactions between neurons to provide an alternative explanation for the spiking that is traditionally attributed to tuning to external variables (see Methods).

Tuning curves can be “explained away” if the other observed neurons provide a better explanation for spiking than the external variables (Fig. 1C). For instance, in a toy network where neuron 1 is tuned to external variables and projects to neurons 2 and 3. Neurons 2 and 3 will appear tuned, despite having no direct relationship to the external world (Fig. 1C, middle). By using the activity of neuron 1 to predict the spiking of neurons 2 and 3, the tuning properties can be explained away by the more direct interactions between neurons (Fig. 1C, bottom). Apparent tuning can appear in any number of network configurations, but given a set of simultaneously recorded neurons the models used here aim to explain spiking as directly as possible. In physiological data, it is unlikely that we are recording from synaptically connected pairs of neurons. The estimated couplings that we observe are likely to be strongly influenced by common input from outside of the recording area and do not necessarily reflect local, recurrent effects. However, tuning curves can still be “explained away” if the activity of the other observed neurons allows better spike prediction.

**Figure 2:**
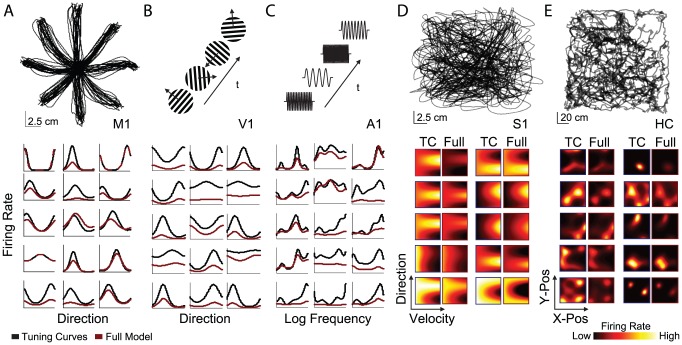
External covariates for each dataset with tuning curve estimates under the tuning curve only and full models. A) Hand position during center-out reaching in a monkey M1 dataset (top) and tuning curves to hand direction for typical M1 neurons under the tuning curve-only model (black) and the full model including coupling between neurons (red). All tuning curve plots are rescaled between zero and the maximum tuning curve value for visualization purposes. B) Randomly oriented gratings for the monkey V1 dataset (top) and typical V1 tuning curves to grating direction. C) Random frequency tones for the ferret A1 dataset (top) and typical A1 tuning curves to frequency fit using radial basis functions in the log-frequency domain. D) Hand position during random-target pursuit in the monkey S1 dataset (top) and typical 2-dimensional tuning curves to hand position and velocity under the tuning curve model and full model. E) Head position during free-foraging in the rat hippocampus dataset (top) and place fields for typical hippocampal neurons under each model. Color in E and D denotes firing rate. The color scale is the same for TC and full, but differs across neurons. Note that, for most neurons, the modulation decreases when coupling to other neurons is included (full model), while preferred direction, velocity, and place remain similar.

We fit spike count data from multi-electrode recordings in 6 different brain areas using *maximum a posteriori* (MAP) estimation for each of the three models (see Methods). Data from motor cortices were recorded during reaching movements to measure tuning to hand direction (Fig. 2A, top). Data from visual cortex were recorded during the presentation of drifting gratings (Fig. 2B, top). Data from auditory cortex were recorded during the presentation of pure tones (Fig. 2C, top). Data from primary somatosensory cortex were recorded during reaching (Fig. 2D, top). Data from hippocampus were recorded during free foraging (Fig. 2E, top). Details of the experiments as well as model fitting and validation procedures are included in the methods.

**Figure 3:**
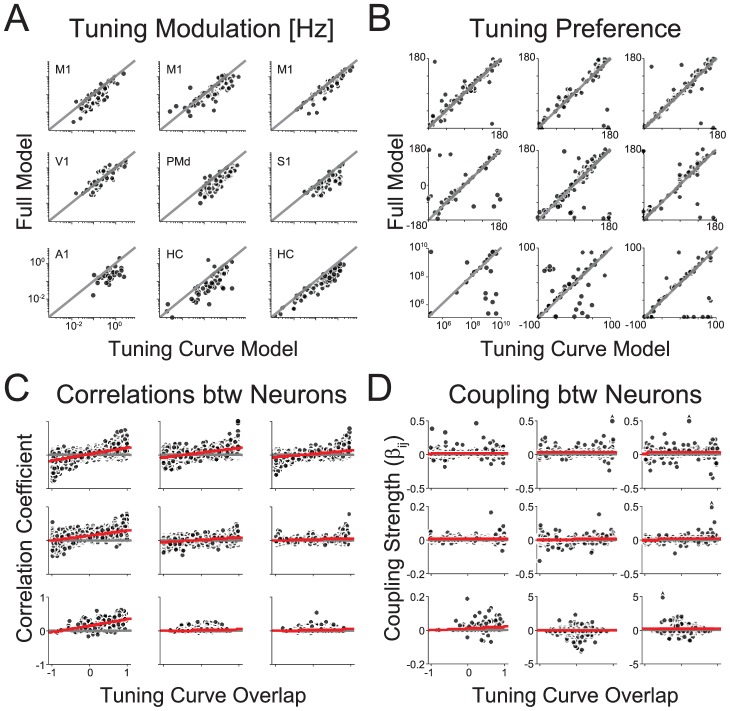
Changes in tuning under the full model and the relationship between coupling, correlation, and tuning curve overlap. A) Tuning curve modulation (max-min) under the tuning curve and full model. Note that the modulation attributed to the tuning curve decreases under the full model on average for all datasets. B) Tuning curve preference under the tuning curve and full model. Preferred direction is shown for M1/V1/PMd/S1 (deg), preferred frequency for A1 (Hz), and preferred place along the x-axis for HC (cm). Gray lines denote equality. C) Correlation coefficient as a function of tuning curve overlap for all pairs of neurons. Note that, in general, correlations increase with increasing tuning curve overlap. D) Coupling strength as a function of tuning curve overlap for all pairs of neurons. Note that, in general, coupling strength does not depend on tuning curve overlap. Red lines denote linear fit. Plots in B–D are organized by dataset as in A.

In the full model, most, but not all, neurons showed decreased modulation to external variables (Fig. 2, bottom). That is, spiking that was previously attributed to tuning properties was more directly explained by functional interactions with other neurons. However, the structure of the tuning curve (i.e. the preferred direction, frequency, or place) remained relatively unchanged. The tuning modulation (minimum-to-maximum) decreased 34–82%, with hippocampus showing the smallest decrease and primary auditory cortex the greatest decrease (Fig. 3A). On the other hand, typical tuning preferences are generally well-preserved (Fig. 3B). Preferred direction, frequency, and place are consistent between the tuning curve model and the full model (correlation coefficient R = 0.34–0.86, circular correlation coefficient where appropriate).

To quantify how coupling in the full model relates to tuning properties we measured the overlap between tuning curves for each pair of neurons in each dataset using the angle between the tuning curve parameter vectors (cosine similarity). An overlap of zero corresponds to orthogonal tuning (i.e. cosine tuned neurons with preferred directions of 0 and 90 deg), an overlap of one corresponds to identical tuning, and an overlap of negative one corresponds to exactly opposite tuning (i.e. cosine tuned neurons with preferred directions of 0 and 180 degrees). We find that tuning curve overlap is clearly related to the bulk spike-count correlation across all stimulus/movement conditions (Fig. 3C). However, coupling strength is only indirectly related to tuning curve overlap (Fig. 3D). Two neurons having similar tuning curves will not necessarily have strong coupling in the full model. This suggests that the explaining away of tuning curves by coupling is not a straight-forward byproduct of stimulus correlation and that including other observed neurons in spike prediction provides information that is not present in the tuning curves alone.

**Figure 4:**
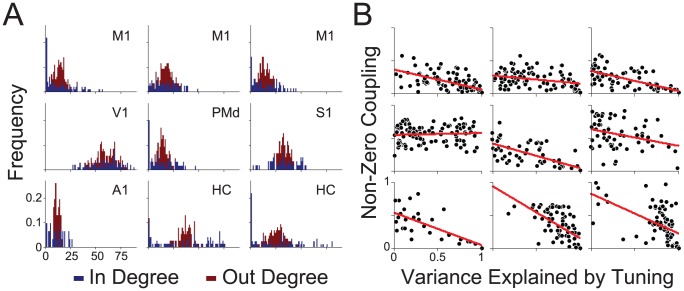
Sparseness and fraction of variance explained by tuning. A) Degree distributions, both in-degree (blue) and out-degree (red), for the coupling matrices estimated from each dataset under the full model. B) The fraction of non-zero inputs for each neuron as a function of the fraction of variance explained by tuning. Red lines denotes the linear trend. Plots are organized by dataset as in A.

The structure of the coupling terms, particularly the number of connections that each neuron makes with the other observed neurons (the “degree”) provides some insight into how tuning curves are explained away. In contrast to theories of scale-free neural connectivity [29] – which predict power-law degree distributions – the estimated functional interactions in these datasets, under the full model, have uni-modal degree distributions (Fig. 4A). Interestingly, across all datasets, it seems that out-degree (how many outputs a neuron drives) is more narrowly distributed than in-degree (how many inputs a neuron receives). The exact structure of the functional connectivity graphs may be affected by electrode spacing and geometry [24]. However, in-degree is correlated with how well coupling can explain tuning (Fig. 4B). In general, neurons whose tuning curves are well explained by coupling receive input from more neurons compared to neurons whose tuning curves are not well explained by coupling.

**Figure 5:**
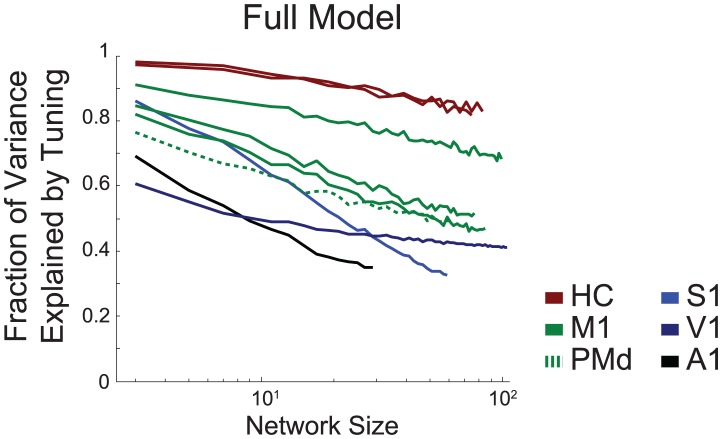
Mean fraction of variance explained by tuning as a function of network size. For the full model the fraction of variance explained by the tuning component of the model decays approximately logarithmically (note the log-scale) to 30–90% of the total variance when all neurons are included in the model.

How these models behave as the number of simultaneously recorded neurons grows is an important consideration for future modeling. Here we fit the coupling alone and the full model, varying the number of neurons used to predict spikes. Under the full model, we find that, in good approximation, the fraction of variance explained by tuning decreases logarithmically as the number of observed neurons increases (Fig. 5). Place fields in hippocampus are explained away slowly, while tuning curves in motor and sensory cortices are explained more rapidly. In general, 10–70% of the variance initially attributed to tuning curves is explained by coupling between neurons in the full model.

**Figure 6:**
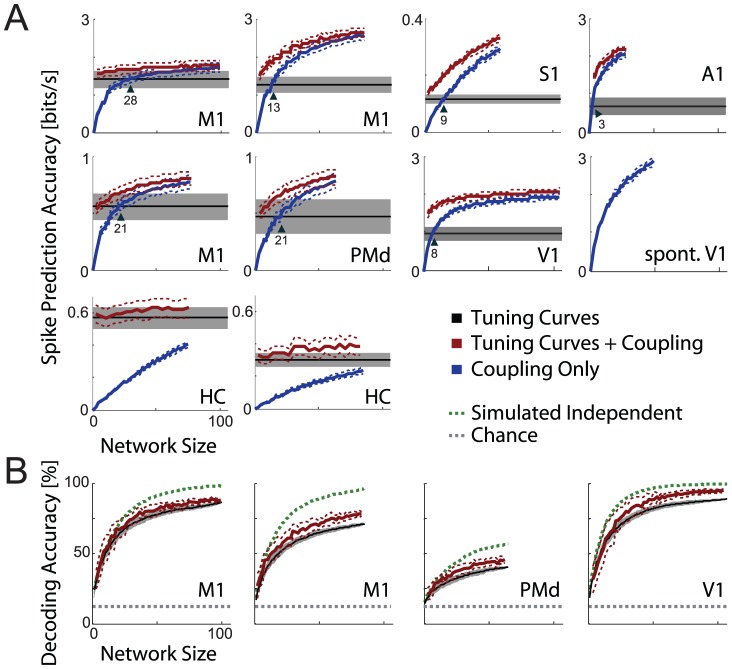
Encoding and decoding accuracy. A) Mean spike prediction accuracy under the tuning curve (black), coupling (blue), and full (red) models. Where spike prediction accuracy denotes the cross-validated log likelihood ratio relative to a homogeneous Poisson process reported in bits/s. Error bars denote SEM across neurons (tuning curve models) or networks (coupling and full models). In all cortical areas the coupling model out-performs tuning curve models once coupling between 10–30 neurons is included. Note that for spontaneous activity in V1, coupling improves spike prediction accuracy even though stimuli were not displayed and tuning curves cannot be fit. B) Mean decoding accuracy under the tuning curve (black) and full (red) models, as well as accuracy for simulated, conditionally independent neurons with data-matched tuning curves (green). Note that the full model slightly outperforms the tuning curve model alone, but dependencies between neurons degrade decoding performance relative to the simulated conditionally independent neurons. Error bars denote standard deviation across neurons (tuning curve models) or networks (full model).

A second metric for studying how these methods scale with the number of observed neurons is spike prediction accuracy (see Methods). As the number of neurons included in the model increases we find that spike prediction accuracy scales, to a good approximation, hyperbolically (Fig. 6A). Note that the full model begins providing the same accuracy as the tuning curve model. As more neurons are included in the model, spike prediction accuracy increases and appears to converge towards a maximum. Interestingly, modeling coupling alone shows this same hyperbolic behavior, beginning at zero and converging towards a maximum. Once 10–30 neurons are included in the model, coupling alone provides more accurate spike prediction that traditional tuning curve models in most datasets.

Hippocampal neurons appear to differ from cortical recordings in that spike prediction accuracy increases approximately linearly. Moreover, modeling coupling alone does not provide more accurate spike prediction than the basic place field model. This may be due to the low correlations between HC neurons. Electrode spacing may also be a factor, since, unlike the 400 µm electrode spacing used in almost all of the intra-cortical arrays, HC recordings had 20 µm vertical electrode spacing. However, the coupling model for spontaneous activity in V1 shows the same hyperbolic behavior despite data being recorded using a polytrode with 50 µm electrode spacing. Scaling of spike prediction accuracy in hippocampus appears to be qualitatively different from that in cortex.

In addition to examining how encoding accuracy scales with the number of recorded neurons, we also examined decoding accuracy for several datasets (Fig. 6B). For the V1, M1, and PMd datasets, we infer which of eight different reach targets or stimuli was presented given the observed spiking on a given trial. Here we use Bayesian decoding under either the tuning curve encoding models or the full encoding model described above (see Methods for details). As with spike prediction accuracy, decoding accuracy grows approximately hyperbolically as more neurons are included in the models. Including coupling between neurons in addition to tuning improves decoding by a small but significant amount: 4.8±0.3%, 7.8±0.4%, 10.3±0.4%, and 7.9±0.3% for the two M1 datasets, PMd, and V1, respectively. Many studies have illustrated how dependencies between neurons can reduce decoding accuracy [30]. By simulating from the tuning curve model we can examine how well we could decode external variables if the neurons were conditionally independent. In this case, decoding from such an independent population of neurons would be ∼25% more accurate than decoding the observed data with the tuning curve model.

**Figure 7:**
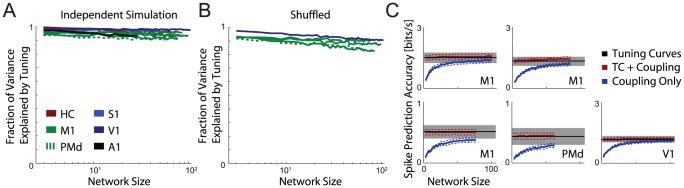
Controls for over-fitting and removing noise correlations using within stimulus/target shuffling. A) Mean fraction of variance explained by tuning as a function of network size for simulated, independent neurons whose tuning curves were matched to the recorded data. The fraction of variance explained by tuning remains close to 1, indicating that there is little to no over-fitting. B) Mean fraction of variance explained by tuning as a function of network size for shuffled data. These results provide an additional control for over-fitting, while retaining stimulus correlations. C) Mean spike prediction accuracy under the tuning curve (black), coupling (blue), and full (red) models on shuffled data. Error bars denote SEM across neurons (tuning curve models) or networks (coupling and full models).

It is important to consider what factors may be driving these scaling phenomena. Although the coupling terms are regularized during estimation and the spike prediction accuracy is cross-validated, it may be the case that tuning curves are explained away as a result of over-fitting or, alternatively, as a simple side effect of stimulus correlations. To test for this possibility we simulated spike counts from the tuning curve model, where the neurons are conditionally independent given the external variables. That is, although there may be stimulus correlations, spiking can be completely predicted by external variables. Here we find that no matter how many neurons are included in the full model, tuning explains between 90–100% of the variance (Fig. 7A). This suggests that the results for the full model in real data are not driven by over-fitting or stimulus correlation alone.

Additionally, we can quantify how much stimulus correlation contributes to explaining away by shuffling the data to remove noise correlations. Where possible (M1, PMd, and V1) we shuffle the spike counts within each trial condition (target or grating direction) independently for each neuron. This manipulation retains stimulus correlations while destroying any structure unrelated to the stimulus. Here we find that, in the full model, tuning explains between 85–95% of the variance (Fig. 7B). Furthermore, the spike prediction accuracies of the full and coupling models do not exceed the accuracy of the tuning curve model in shuffled data (Fig. 7C). These two controls demonstrate that the observed explaining away is not simply a byproduct of stimulus correlations or of a poor tuning curve model. Explaining away can only occur when the other observed neurons provide a more direct explanation of spiking than the external variables.

**Figure 8:**
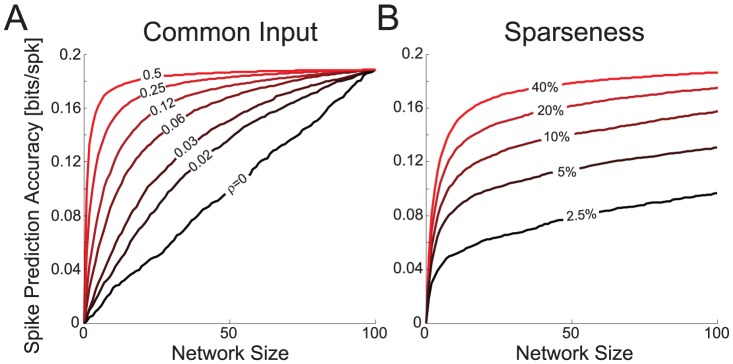
Scaling of spike prediction accuracy as a function of common input and sparseness. A) Spike prediction accuracy for simulated neurons receiving correlated input, with different levels of input correlation. For these simulations all neurons contribute to the output (100% non-zero entries). B) Spike prediction accuracy for simulated networks with different input sparseness. For these simulations the input correlation is fixed at 0.25. Percentages denote the fraction of non-zero input.

Finally, to examine what drives the shape of these spike prediction accuracy curves we simulated a linear-nonlinear-Poisson neuron receiving sparse, correlated input. As input correlation increases spike prediction accuracy converges more quickly to its maximum (Fig. 8A). When the inputs are strongly correlated, neurons added later are only providing redundant information. However, when the inputs are independent, each additional neuron contributes to more accurate spike prediction. If the inputs are sparse and some of them are irrelevant to the prediction, information added by each neuron is simply smaller on average (Fig. 8B). That is, if only 10% of the inputs are non-zero then it takes 10 times as many neurons to reach a given spike prediction accuracy compared to the case where all of the inputs were non-zero. For input correlation 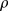 and probability 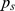 of a given input being non-zero, the simulations are well-approximated by a hyperbolic function 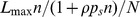 where 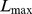 is the maximum spike prediction accuracy and 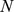 the maximum number of neurons.

Linking the strength of common input and sparseness to the spike prediction accuracy curves observed in real data is difficult. Both a weakly correlated, highly connected network and a highly correlated, highly sparse network will have near-linear growth. However, here we find that neurons in cortex (particularly V1 and A1) tend to be more strongly correlated than neurons in hippocampus (Fig. 3C). This may partially explain the rapid growth in spike prediction accuracy for the cortical datasets and, in comparison, the near-linear growth for hippocampal datasets. Especially in cortex, the fact that neural activity traditionally attributed to tuning curves is more directly explained by interactions between neurons appears to be a byproduct of unobserved common input.

## Discussion

Excepting peripheral neurons such as photoreceptors, the relationship between a neuron's spiking and the external world is a result of the input that each neuron receives from other neurons. Many studies have examined how pre-synaptic input determines receptive field structure and tuning properties both experimentally [e.g. 1], [3], [4], [5] and theoretically [31]–[33]. Here we have used multi-electrode recordings and statistical modeling to examine, broadly, how tuning curves might be explained, in a statistical sense, by functional interactions between neurons. We have found that, in a variety of brain areas, modeling coupling between a relatively small number of simultaneously observed neurons in the same brain area allows more accurate encoding and decoding. As the number of observed neurons grows the fraction of spiking variability attributed to tuning appears to decrease logarithmically, while spike prediction accuracy increases hyperbolically. Once interactions between 10–30 neurons are modeled, coupling alone can often provide more accurate spike prediction than traditional tuning models.

The extent to which the activity of simultaneously observed neurons or tuning properties explain spiking likely depends on a number of factors including the timescales on which we model spiking, the stimulus or task parameters, and the external variables being used to describe tuning. The coarse, instantaneous coupling models used here cannot distinguish between the many possible hidden causes of correlated neural activity. Since the models used here reflect pair-wise dependencies on a long timescale of 100 s of milliseconds, it is likely that unobserved behavioral variables and internal processes make strong contributions to the coupling terms. Modeling these effects explicitly may yield a more nuanced view of the relationship between tuning curves and interactions between neurons [23], [34]. Although we model trial-by-trial spike-count data here, both tuning and coupling can also be modeled as history-dependent effects. Since external variables change fairly slowly, whereas interactions between neurons are likely to be relatively fast, adding such temporal information may result in qualitatively different results. More detailed models that include history-dependent coupling on millisecond timescales may be able to further unpack the functional roles of recurrent, local coupling and instantaneous common input [35].

The datasets used here yield surprisingly similar results considering that they were recorded from different brain areas and species with different electrode configurations. However, in addition to these anatomical differences, it is important to note that the datasets were recorded under a variety of experimental circumstances, which may help to explain some of the remaining differences in the results obtained from each dataset. For data from motor cortices and hippocampus, for instance, the external variables are not controlled in the same way that sensory experiments are. Movement variables such as velocity or body orientation differ even when the monkey's reaches to the same target or when the rat is at the same maze location. These external differences may lead to higher apparent trial-to-trial variability. Additionally, data from V1 was recorded while the animals were under anesthesia, which may lead to higher correlations between neurons [36]–[38].

The tuning models used here, despite their wide-spread use, are relatively simplistic. Tuning functions that take into account more external variables are likely to give more accurate spike prediction, and including these variables may change the degree to which tuning properties are explained away as interactions between neurons are added to the model. At the same time, exploring the space of external variables and determining what causes a neuron to fire can be difficult [39]–[41]. Fitting tuning functions to neurons in medial temporal lobe, for instance, might require exploring the space of all possible objects [42]. Fitting high-dimensional tuning functions, in general, can require large amounts of data as well as sophisticated estimation methods [43]. Rather than exploring the space of external variables, exploring the statistical structure of interactions between neurons may be an alternative strategy for understanding tuning properties.

### The unreasonable effectiveness of small numbers of neurons

Neurons receive pre-synaptic input from tens of thousands of other neurons, and each of these inputs, presumably, plays a role in determining the tuning properties of a post-synaptic neuron. How is it possible then that models of interactions between <100 neurons are able to explain spiking more directly than traditional tuning curve models without any guarantee that the neurons are even anatomically connected?

Ultimately, explaining away can only occur when neural activity is not independent. Many studies have examined correlated neural activity [38], [44]–[48] as well as its potential functional roles [30], [37], [49], [50]. Here correlations between neurons are essential in allowing tuning properties to be explained away by the functional interactions between small numbers of neurons. However, the fact that coupling terms do not explain away tuning curves in simulated or shuffled data, suggests that our results are not simply a byproduct of stimulus correlation. Rather, the estimated coupling between neurons is likely to reflect a combination of direct and indirect interactions [e.g. 51] as well as additional unobserved common input [14] and internal processes [52]–[54]. Several studies have made progress in attempting to infer unobserved common input related to the external world [34] as well as internal processes [35], [55]. Here we simply note that unobserved common input may allow more accurate spike prediction in models of interacting neurons by creating correlations that cannot be attributed to the observed external variables. Modeling these dependencies improves decoding by a small, but significant, amount and may be useful for improving brain-machine interfaces [56]. Moreover, the correlations induced by unobserved common input appear to allow neural activity traditionally attributed to tuning properties to be more directly explained by interactions between neurons.

It is important to note, however, that the statistical approaches used here are unlikely to capture anatomical information about the underlying circuitry. These methods still only provide a sketch of the underlying circuit that best explains the observed spiking. The hyperbolic scaling of spike prediction accuracy observed here, for instance, may be a general property of correlated prediction problems [57]. found a similar hyperbolic scaling in accuracy using the firing rates of neurons in motor cortex for linear prediction of hand position.

### Towards a description of tuning properties based on network architecture

For many years, studies of the relationship between neural interactions and tuning properties have been based on detailed electrophysiology [58], [59], experimental intervention , or simulation [32], [60]. Most of these studies have addressed data collected in sensory cortices or peripheral areas. However, understanding the response properties of neurons in other areas, such as motor and association cortices, in terms of neural circuits has been difficult. Here we used simultaneous neural recordings and a model-based statistical approach to ask how well tuning properties can be explained, in a statistical sense, by functional interactions between neurons. While these models are able to explain a surprisingly large fraction of the variation in neural spike counts in a variety of brain areas with a relatively small number of observed neurons, they only provide a rough picture of how network *architecture* might give rise to commonly observed tuning properties.

Understanding how interactions between neurons give rise to tuning properties, will ultimately mean understanding the relative contributions of feed-forward, local, and top-down pre-synaptic inputs, as well as how different subtypes of neurons and neurons with different types of tuning interact. One area where statistical approaches have revealed this type of detailed architecture is in the retina. By recording from dense populations of retinal ganglion cells (RGCs), recent work has shown that RGC receptive fields arise directly and clearly from input received from rods and cones [61]. Moreover, functional interactions between retinal ganglion cells appear to have a strong, local structure [21]. Although photoreceptors are the only elements in the retinal circuit that have direct responses to the external world, the receptive fields of RGC responses can be understood as a byproduct of indirect interactions with photoreceptors, mediated by intermediate neurons, such as horizontal, amacrine, and bipolar cells.

In most areas of the brain, beyond the retina, recording from a complete neural circuit is experimentally infeasible and the complete network of neurons is immensely under-sampled. In these cases, it is difficult to determine whether potential interactions between neurons are direct (mono-synaptic) or indirect (poly-synaptic), and the estimated interactions are likely to be strongly influenced by unobserved common input [14]. What is ultimately estimated by the statistical approaches is a phenomenological model of the circuitry that best describes the observed spikes [20]. For this reason it is difficult to draw conclusions about detailed architecture in current multi-electrode datasets. Here we have examined how modeling interactions between small numbers affects neural coding and how model-based estimates of interactions relate to stimulus and noise correlation. As electrophysiologists record from increasing numbers of neurons [13] these approaches have the potential to reveal more detailed information about the structure of these cortical and sub-cortical areas.

## Methods

We analyzed 10 multi-electrode spike datasets recorded from 6 different brain areas and 4 different species. Recordings from primary (M1) and dorsal pre-motor cortex (PMd) were made while a macaque monkey performed a center-out reaching task. Recordings from primary sensory cortex (S1) were made while a macaque monkey performed a random-target pursuit task. Recordings from primary auditory cortex (A1) were made while a ferret was exposed to random frequency tone stimuli. Data from primary visual cortex (V1) consisted of recordings of 1) evoked activity while an anesthetized monkey viewed randomly oriented moving gratings and 2) spontaneous activity from an anesthetized, paralyzed cat. Finally, recordings from dorsal hippocampus (HC) were made while a Long-Evans rat was freely foraging for food on a square platform.

All animal use procedures were approved by the institutional animal care and use committees at Northwestern University (M1 & S1), University of Chicago (M1 & PMd), Albert Einstein College of Medicine (V1), University of Maryland College Park (A1), University of British Columbia (V1 spont), or Rutgers University (HC) , and conform to the principles outlined in the Guide for the Care and Use of Laboratory Animals (National Institutes of Health publication no. 86-23, revised 1985). Data presented here were previously recorded for use with multiple analyses. Procedures were designed to minimize animal suffering and reduce the number used.

The aim of our analysis was to examine the relationship between typical tuning curves and receptive fields in each of these brain areas and coupling between neurons. To this end we extracted spike count data from the spike-sorted multi-electrode recordings and focused either on evoked responses for the stimulus and directed movement tasks or binned responses for the foraging and spontaneous tasks. Each dataset contained at least 31 and as many as 107 simultaneously recorded, putative single neurons after spike sorting (Table 1).

**Table 1:** Summary of the datasets used here.

Area	Neurons	#Trials/Bins	Bin Size (ms)	Stimulus/Task
M1	87	290	200	Center-out Reaching
M1	101	193	200	Center-out Reaching (Wrist)
M1	78	315	200	Center-out Reaching
PMd	65	315	200	Center-out Reaching
S1	61	3539	200	Random Target Pursuit
V1	107	3200	400	Drifting Sine-Wave Gratings
V1	50	3600	100	N/A (Spontaneous)
A1	31	165	100	Pure Tones
HC	76	5000	250	Free Foraging
HC	87	5000	250	Free Foraging

### Multi-electrode recordings

#### Recordings from primary and pre-motor cortex

Datasets were obtained from the motor cortices of two monkeys (designated K and R). Monkey K was implanted with a 100-electrode Utah array (Blackrock Microsystems, 400 µm spacing, 1.5 mm length) in the arm area of primary motor cortex. Data were recorded during two different tasks: a standard eight-target center-out reaching task, and an isometric eight-target center-out wrist force task. In the first task the monkey was seated in a primate chair, with movement constrained to a horizontal plane, with the arm roughly in a sagittal plane. The monkey grasped the handle of a two link planar manipulandum that moved within a 20 cm by 20 cm workspace. In the second task the monkey used isometric forces about the wrist (with the forearm in a posture midway between pronation and supination) to produce center-out forces. In both tasks feedback about movement or force was given on a computer screen in front of the monkey, displayed as a circular cursor, 1–2 cm diameter. The recordings were made approximately 4 months apart with 87 well isolated single-units recorded during the center-out reaching task and 101 units recorded during the center-out wrist-force task after offline spike-sorting. Trial-by-trial spike counts were collected during the period 100–300 ms following movement onset on 209 and 193 trials, respectively.

For Monkey R, two 100-electrode Utah arrays (Blackrock Microsystems) were implanted in dorsal pre-motor and primary motor cortices. Data were recorded while the monkey performed a randomized, eight-target, center-out reaching task using a KINARM device (BKIN Technologies, Kingston, ON, Canada) in which the monkey's arm rested on cushioned troughs secured to links of a two-joint robotic arm [62]. After spike-sorting, 78 well isolated single-units were recorded in M1 with 65 well isolated single-units in PMd. Data from M1 and PMd are treated separately here, and trial-by-trial spike counts were collected during the period 100–300 ms following movement onset from 315 trials. See [63] for surgical, stimulus, and preprocessing details.

In each of the M1 and PMd datasets neuronal signals were classified as single- or multi-unit based on action potential shape and minimum inter-spike intervals greater than 1.6 ms. Spike sorting was performed by manual cluster cutting using an offline sorter (Plexon, Inc). All trials for the center-out tasks began with the acquisition of a square center target that the monkey was required to hold for 0.3–0.5 s. Subjects had 1.25 s to acquire the peripheral target and were required to hold this outer target for at least 0.2–0.5 s. Each success was rewarded with juice or water.

Datasets are available for download at http://crcns.org/data-sets/movements/dream.

#### Recordings from primary somatosensory cortex

One dataset was recorded from a macaque monkey implanted with a 100-electrode Utah array (Blackrock Microsystems) in primary somatosensory cortex (areas 1 and 2) while the monkey performed a random-target pursuit task. The monkey was seated in a primate chair, with movement constrained to a horizontal plane. The monkey grasped the handle of a two link planar manipulandum that moved within a 20 cm by 20 cm workspace. After each target hit a new target would appear in a random location. A run of 3–4 successes was rewarded with juice or water.

Neuronal signals were classified as single- or multi-unit and spike sorted as above to provide 61 well-isolated units. Trial-by-trial spike counts were collected during the period 100–300 ms following movement onset from 3539 trials for subsequent analyses. See [64] for surgical, experimental, and preprocessing details.

#### Recordings from primary auditory cortex

Data from the primary auditory cortex A1 of an awake, passively listening ferret were recorded during presentations of pure-tones of various frequencies spanning 6.4 octaves (0.18–15.56 kHz) equally spaced in log-frequency presented in random order over 165 trials. Recordings were made with a 32 electrode array (500 µm spacing, 2.5 MΩ, Microprobes Inc.) in Layer IV (depth ∼700 µm). Neuronal signals were classified as single- or multi-unit based on action potential shape and inter-spike intervals greater than 1.6 ms. Spike sorting was performed by manual cluster cutting, providing 31 well-isolated units. Trial-by-trial spike counts were collected during the 100 ms stimulus period. See [65] for experimental details regarding surgery and stimulus presentation.

#### Recordings from primary visual cortex

Two datasets were obtained from primary visual cortex. The first dataset was recorded while an anesthetized monkey viewed one of eight randomly oriented, drifting sine-wave gratings. Stimuli had a spatial frequency of 1 cyc/deg, drift rate of 6.25 cyc/s, size of 2.9 deg and were presented for 400 ms with a 800 ms delay between stimuli. Recordings were made using a 100-electrode Utah array, 400 µm electrode spacing (Cyberkinetics Neurotechnology Systems). After automatic spike sorting and manual cluster adjustment, 107 single units and small multi-unit clusters with firing rates >1 Hz were used. Trial-by-trial spike counts were collected for the entire 400 ms stimulus period for 3200 trials (400 repetitions for each orientation) for subsequent analyses. See [44] and [66] for surgical, stimulus, and preprocessing details.

The second dataset from primary visual cortex was downloaded from the Collaborative Research in Computational Neuroscience (CRCNS, http://crcns.org) data sharing initiative [67]. Briefly, recordings consisted of spontaneous activity from well-isolated units in area 18 of an anesthetized, paralyzed cat. Recordings were made using a 54-channel polytrode, 50 µm vertical separation [68]. The recording was approximately 6 min in duration with 50 well isolated neurons after semi-automatic spike sorting and manual verification. Spike counts were binned over 100 ms intervals for subsequent analyses. See [68] for implantation, experimental, and preprocessing details.

#### Recordings from dorsal hippocampus

Two hippocampal datasets were obtained from the Collaborative Research in Computational Neuroscience (CRCNS, http://crcns.org) data sharing initiative (data collected in the Buzsaki lab; http://www.med.nyu.edu/buzsakilab/). Briefly, recordings consisted of well-isolated units in the right dorsal hippocampus (CA1) of two Long-Evans rats (250–400 g). Recordings were made using an 8-shank silicon probe, each shank with 8 recording sites, 20 µm vertical separation [69], while the rat foraged for water rewards on an elevated platform (180 cm×180 cm). Recordings were approximately 90 min in duration with 76 and 87 well isolated neurons after automatic spike sorting and manual cluster adjustment. Spike counts were binned over 250 ms intervals for subsequent analyses. See [70] for implantation, task, and preprocessing details.

### Data analysis

Spike count data were fit using either external variables, the activity of the other recorded neurons, or both [14], [17], [21], [28]. In each case we used a class of generalized linear model [71] - a linear non-linear Poisson (LNP) model with exponential nonlinearity [15], [16]. The model and estimation methods have been previously described in detail elsewhere [21]. Briefly, LNP models assume that the covariates (tuning to stimulus/movement or activity of other neurons) are linearly combined, then passed through an exponential nonlinearity such that the firing rate is non-negative. The estimated firing rate for each neuron 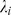 is then a function of the external variables during each trial 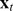 and the activity of the other neurons 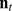:
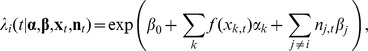
where 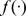 denotes one of 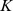 basis functions that describe the shape of the tuning curve, and the parameters 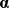 and 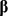 capture tuning, coupling to other neurons, and a baseline firing rate 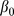. The basis functions, described below, will depend on the brain area we are trying to model and the stimulus/task. The spike count is then assumed to be drawn from a Poisson distribution with this rate:
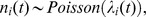
where 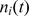 represents the spike count for neuron 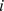 on trial 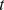.

Using this same framework, tuning curves alone were modeled by
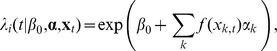
while coupling between neurons was modeled by
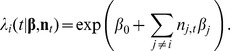



Note that, for the coupling model, the spike count for the neuron 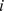 whose firing rate we are estimating was always excluded. Using this framework we examined the effect of network size on spike prediction accuracy by varying the total number of neurons included in the model and using a random subset of all recorded neurons, again excluding neuron 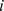.

For each of these three models – the full model, tuning curve model, and coupling model – we estimated the parameters 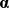 and 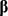 directly from the observed spike count data using maximum likelihood estimation (MLE) or maximum *a posteriori* (MAP) estimation with an L1-penalty to prevent over-fitting [see 21]. Here we compute ML estimates using iterative reweighted least squares (IRLS) with the Matlab package *glmfit* and compute MAP estimates using path-wise, cyclical coordinate descent [72] with the R package *glmnet*.

Where regularization is used we optimized the regularization hyperparameter via the cross-validated (10-fold) log-likelihood, and in all cases we evaluated the “spike prediction accuracy” of the models using the cross-validated log likelihood ratio relative to a homogeneous Poisson process. For a firing rate 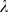, the log-likelihood is given by
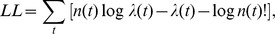
and the log likelihood ratio relative to a homogeneous Poisson process (spike prediction accuracy) is given by
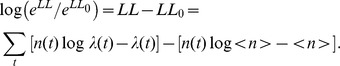



In this case, a spike prediction accuracy of zero corresponds to a model that does no better than predicting the mean spike count. Values were calculated in base-2 and rescaled by time to give units of bits/s [see 21,27]. We find that spike prediction accuracy scales approximately hyperbolically, following 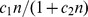 where 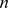 is the number of neurons in the model and 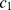 and 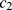 are parameters determining the shape of the curve [see 57].

An important component of these models is the choice of basis functions for the external variables. Here we have attempted to choose common tuning models, appropriate for each dataset. For M1 and PMd neurons, for instance,
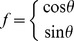
where 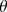 denotes the target direction on each center-out trial. This linear component of the model corresponds to the traditional cosine tuning models of motor cortical neurons [73], [74]. We used the same model to capture direction tuning in visual cortex [75].

While activity in M1 has also been shown to covary with speed [76], we elected to use the simpler direction tuning model here, and model speed tuning only in S1 neurons, which show direction tuning [77] as well as clear tuning to hand speed [64]. In this case we use
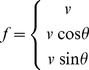



Place fields of the neurons in hippocampus have been well described [78]–[80]. To model these localized response properties we use a set of radial basis functions that tile the foraging area.
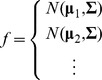



Specifically we use K = 25 isotropic Gaussian radial basis functions equally spaced on a 5×5 grid with means 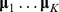 and covariance 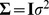, 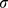 = 9 cm.

Finally, for neurons in A1 [81]–[83], we again use radial basis functions. In this case K = 7 Gaussians were equally spaced along the log-frequency of the stimulus with standard deviation 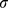 = 0.64 octaves.

In most cases (TC dimensionality <4), regularization was only applied to the coefficients modeling coupling between neurons. To avoid convergence problems [84] the models using radial basis functions (A1 and HC) included weak regularization on the tuning curve coefficients (with 20% of the L1-penalty used for the coupling coefficients). The tuning curve parameters do not change substantially for penalties ranging from 1–20%; however, there may be unintended shrinkage in these models, and the decrease in modulation observed for these neurons may be somewhat over-estimated.

It is important to note that the models used here differ from previous approaches in that they are time-instantaneous — we model coupling between neurons at the same time. This does not pose any difficulties during fitting, since we are modeling only the conditional distributions for each neuron 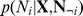. However, simulating from the joint spiking distribution 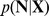 is no longer straight-forward. The usual assumption, 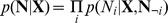, does not hold, but, since the conditionals are know, we can use Gibb's sampling to simulate from this joint distribution if necessary (see section on Decoding below).

To quantify the changes in tuning under the full model we evaluate the tuning modulation, tuning preference, and tuning curve overlap between pairs of neurons. Tuning modulation is simply the peak-to-peak difference in firing rate for the tuning curve component of the model, reported in Hz. Tuning preference is defined differently for each dataset: for M1, V1, PMd, and S1 we use the preferred direction, for A1 we use the preferred frequency, and for HC we use the preferred place along the x-axis. Finally, to measure similarity between the tuning curves for pairs of neurons we evaluate the tuning curve overlap between neurons 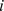 and 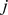, 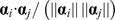. Accordingly, a tuning curve overlap of 1 suggests that the two neurons have identical tuning (up to a constant baseline), while a tuning curve overlap of 0 suggests that the two neurons have orthogonal tuning.

To quantify network properties we also report the spike count correlation (Pearson's correlation). For two neurons with trial-by-trial spike count observations 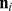 and 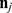 the correlation is given by 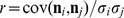. Although the spike count correlation between pairs of neurons is known to increase with both firing rate [85] and time interval [86], [87], we do not attempt to correct for these effects here. In most cases the bin-size is determined by the task and the traditional periods used to measure tuning curves, such as stimulus duration.

To quantify the relative contributions of the tuning curve and coupling components in the full model we summarize the fit using the fraction of variance explained by tuning. For each neuron we calculate
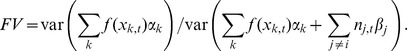



A value of 1 suggests that the coupling terms provide no additional information, while a value of 0 suggests that any tuning information is explained completely by coupling to other observed neurons. It is important to note that there is considerable heterogeneity in how well tuned neurons are to the external variables. Here we analyze all recorded neurons, even those that might be considered un-tuned.

### Decoding

In contrast to the encoding models above, which aim to predict spikes given a stimulus, we can also examine how coupling affects decoding, which aims to predict a stimulus 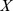 given a set of spike observations 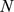. Here, we use Bayesian decoding [88], [89] based on the same encoding models described above. Assuming the stimuli are equally probable
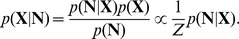



For the tuning curve models, we assume that the neurons are conditionally independent given the stimulus,
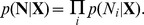



However, for the full model, since we assume that coupling is instantaneous, we cannot make this assumption. In this case we use a variation of Gibb's sampling [90]–[92] to approximate the joint distribution 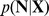. Briefly, in Gibbs sampling we generate samples from 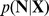 by iterating over all neurons and sampling a spike count for each neuron based on the conditional distribution
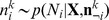
where 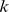 denotes the iteration. Here we use a method know as ordered over-relaxation [93] to improve mixing. This makes the sampler much more efficient for large networks of neurons with strong coupling. In this case we generate 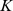 samples from the conditional distribution at each update, sort the samples along with 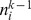, and if 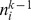 is the 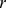-th largest value we take the 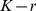-th sample. After many iterations the set of samples - ignoring a burn-in period - provides an approximation to the joint likelihood that we can then use, via Bayes rule, to approximate the posterior over possible stimuli.

For each model we initialize the sampler with 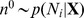, initialize each successive sample using 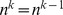, and update the spike counts 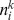 for each neuron in a random order using the conditional distribution 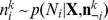 with ordered over-relaxation. We then take 5000 samples after a burn-in period of 500 samples to use as an approximation to the joint density.

In practice, it is non-trivial to estimate the probability 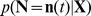 for each trial given a set of samples from the distribution 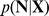. When the number of neurons become large the curse of dimensionality makes histogram estimation impossible. We would need 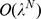 samples where 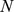 is the number of neurons to construct an accurate histogram. Here we use an approximation based on the chain rule of probability
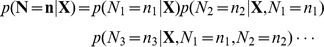



Although we cannot write down the full joint probability analytically, we can approximate each of the marginal distributions in the chain rule using the set of Gibbs samples
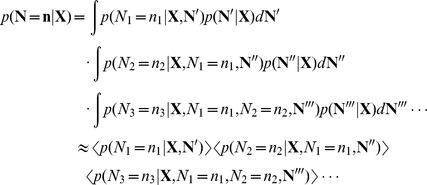
where 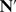 denotes the random variables for all other neurons not yet taking a specific value 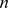, and the expectations 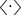 are taken over the set of Gibbs samples. Importantly, each probability in each expectation can now be evaluated analytically based on the conditional Poisson likelihoods of the full model. This approach allows us to approximate the posterior over stimuli and assess the Bayesian decoding accuracy of the tuning curve model with instantaneous coupling.

### Simulations

To examine how the scaling of spike prediction accuracy relates to the underlying structure of the inputs we simulated spikes from a linear-nonlinear-Poisson neuron receiving correlated input
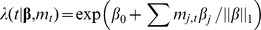


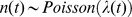
where the baseline firing rate parameter 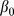 was fixed and 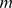 denotes a set of correlated Poisson random variables. The connection strengths 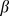 were drawn from a sparse, binary random vector with entries randomly set to zero with probability 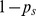. Correlated inputs 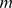 were each assumed to have mean 1 and were drawn from a multivariate Poisson distribution with the covariance matrix 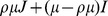 where 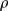 denotes the specified correlation, 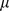 denotes the mean, 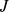 denotes the unit matrix, and 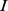 the identity matrix. Under this covariance matrix all pairs of neurons have correlation 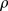 and we set the variance of each neuron equal to the mean.

In general, producing correlated Poisson random variables with specific marginal distributions and covariance structure is difficult. Here we use a simplified family of covariance matrices where all neurons have the same correlation and simulate spike counts following [94]. After simulating and fitting the LNP model, we can examine how input correlations and sparseness affect spike prediction accuracy.
